# Supplementary material for: Are carbon emissions trading and green financial instruments synergistic? -Comprehensive quantitative research based on content analysis
Source: PLoS One. 2024 Mar 7;19(3):e0298601. doi: 10.1371/journal.pone.0298601 (PMC10919601; doi:10.1371/journal.pone.0298601)
Supplement: S1 Data — (ZIP) [file pone.0298601.s001.zip › Data/地级市Z_F工作报告.zip!/║■▒▒/╢⌡╓▌╩╨/└┤╫╘╣1⁄2╓┌║┼í╛┬φ┐╦╩2╛▌═°í┐_2005.docx]

各位代表：

　　现在，我代表市人民政府向大会作政府工作报告，请予审议，并请市政协各位委员和列席人员提出意见。

一、2004年工作回顾

2004年，是我市改革开放和现代化建设取得显著成就的一年。在省委、省政府和市委的正确领导下，全市上下坚持以科学发展观统揽全局，抢抓机遇，克难奋进，开拓创新，扎实工作，经济和社会呈现良好的发展态势，市五届人大一次会议确定的主要目标全面超额完成。

（一）发展速度明显加快。坚持以经济建设为中心，牢牢把握发展这个第一要务，推动经济建设驶入快车道，成为2004年工作的主要特点。

经济总量快速提升。2004年，全市完成生产总值141.9亿元，比上年增长11.8%。其中第一产业增加值18.9亿元，比上年增长6.1%；第二产业增加值74.1亿元，比上年增长13.3%；第三产业增加值48.9亿元，比上年增长11.8%。实现社会消费品零售总额54.53亿元，比上年增长13.6%。全社会用电量达26.2亿千瓦时，比上年增长12.6%，其中工业用电量22.9亿千瓦时，比上年增长14.5%。

固定资产投资再创新高。全年完成固定资产投资44.3亿元，比上年增长18.1%，是1995年以来增长最快的一年。项目建设形势喜人，计划安排60个重点项目中，工业项目占68%。过亿元的15个，总投资均为12亿元的鄂钢二期技改工程和武钢球团矿厂项目已成为我市经济增长的新亮点。

农业发展形势喜人。农业和农村经济出现多年少有的好形势，“政策好、粮价高、人努力、天帮忙”成为2004年“三农”工作的新特点。全年农林牧渔业总产值达18.9亿元（不变价），比上年增长12.4%，主要农产品全面丰收。粮食总产量27.83万吨，比上年增长18.7%；棉花总产量3152吨，比上年增长25.2%；油料总产量5.34万吨，比上年增长6.3%；水产品总产量16.1万吨，比上年增长24.2%；蔬菜总产量74.7万吨，比上年增长8.9%；牲猪出栏66.06万头，比上年增长6.5%。50万亩无公害水产板块建设进展顺利，8个万亩无公害蔬菜基地基本形成；6个养殖小区建设初具规模，防治禽流感工作成效明显。10个花卉苗木基地建设取得新的进展。长江大堤加固工程全面竣工，农田水利基础建设取得新的成绩，农产品加工和流通受到广泛地重视。

开放引进成效显著。全年完成招商引资额2.5亿美元，比上年增长38.8%；实际利用国外境外资金5926万美元，比上年增长18%；外贸出口总值完成2.66亿美元，比上年增长14.9%；出口海关数5612万美元，比上年增长13.1%。成功举办第五届梁子湖捕鱼节，全年接待国内外游客112万人次，实现旅游收入6.72亿元。

（二）经济效益显著提高。坚持以提高经济运行质量为重点，在加快经济发展的同时，保持速度、效益同步增长。

财政收入突破10亿元大关。全年财政收入达到10.5亿元，比上年增长23.6%，是1995年以来增幅最大的一年；完成地方财政一般预算收入4.2亿元，比上年增长24.2%；财政收入结构进一步优化，国地两税占财政收入的比重达89.7%，其中国税收入增长34.2%，地税收入增长7.6%。

工业经济质量不断改善。全市规模以上工业累计完成工业增加值56.2亿元，比上年增长20.2%；实现销售收入173.9亿元，比上年增长22.5%。骨干企业支撑明显，全市重点企业共上交工商税收70487万元，占全市的74.8%；鄂钢和鄂州电厂税收过亿元，市供电公司、程潮铁矿、吴城钢铁、波尔亚太、世纪新峰、华明实业、鄂州烟草公司7家企业税收过千万元。

城乡居民收入大幅增加。农民人平纯收入达3234元，较上年增加402元，增长14.2%，是1995年以来增加最多的一年，比全省平均增量高出69元。农民人平负担下降43.4%。城镇居民人均可支配收入达7490元，比上年增长8.4%。

金融保险业继续发展。信贷质量不断提高，我市被评为A级信用城市。金融机构各项存款余额95.2亿元，比年初增加4.8亿元；贷款余额74.3亿元，比年初增加2.4亿元；保险业得到快速发展，各项保费收入达到1.83亿元，比上年增长12.5%。

（三）发展环境日益改善。坚持以优化环境为突破口，努力改善软硬环境，为可持续发展构筑良好平台。

城市建设规模不断扩大。作为全省人口最少、面积最小的中等城市，如何快速扩大城市规模，提升中等城市地位，是政府工作的重大任务。2004年确定的“一湖五桥九路”工程建设顺利。实施市政公用设施建设项目26个，完成投资额1.7亿元。新建城市道路10条，延伸城市道路17.3公里，沿江大道全线贯通，改造江碧路9.3公里。完成滨湖东路、滨湖南路延伸路基工程，拓展了城东道路骨架。维修改造老城区道路7条，面积达9.8万平方米，铺设人行道彩砖3.5万平方米。建成沿湖游园3个，完成环湖道路护岸5公里。月河桥整险加固工程顺利完成，鄂钢铁路立交桥工程业已开工。农村城镇化建设共投资3071万元。城区道路、园林绿化、给排水等基础设施得到加强。实施交通十大工程，完成建设投资1.91亿元，建成等级公路路基99.21公里、路面53.24公里，农村水泥路面180公里，乡村水泥路覆盖率居全省之首。鄂州一级中心客运站顺利开工，城乡电网、通讯网功能进一步完善。

生态环境建设逐步加强。我市作为全国生态示范试点城市，七大建设工程的63个建设项目责任落实到位；首届梁子湖环境保护论坛成功举办，洋澜湖治理力度不断加大；重点污染源限期治理工作取得成效，对21个建设项目实行了环境影响评价一票否决。城区公共绿地面积25.7万平方米，绿化覆盖率37.6%。国土资源工作取得新成效，非法用地和乱占耕地问题得到查处，经营性土地全部进入市场交易，在全省率先实行探矿权竞价出让。

武汉城市圈建设初现端倪。按照省委、省政府的战略部署，我市出台了《关于加快融入武汉城市圈的实施意见》，从交通、能源、信息、旅游、市场一体化建设等方面。制定了融入武汉城市圈的初步措施，已签约项目11个，协议资金13.9亿元，拟建设的连接东湖开发区与葛店开发区的药谷大道和汉鄂高速公路已进入前期准备阶段。

行政服务环境不断优化。市直14家职能部门194个服务项目进入行政服务中心，使中心的窗口单位达到34家、服务项目436项。坚持实行企业“宁静日”制度，规范了行政执法行为，遏制了对企业正常生产经营活动的干扰。综合招投标中心、政府采购中心已正式运行。政务、村务公开取得新的成效。坚决打击制售假冒伪劣商品等不法行为，市场秩序明显好转。

（四）各项改革稳步推进。坚持以改革为发展动力，把改革的力度与经济发展的速度、社会可以承受的程度结合起来，推进各项事业稳步发展。

企业改革步伐加快。去年是企业改革难度较大、遇到难题较多、问题解决较好的一年。通过一年的攻关，10家特困企业改制工作基本到位、8家即将到位；全年按政策规定减免各类税费2915万元、核销财务损失2.48亿元，并拿出2510万元对特困企业职工投保安置。国有资产管理体系逐步完善，资产经营工作正在探索新的有效方式。

财政改革成效显著。部门预算、国库集中收付、政府采购、收支两条线等四项财政制度改革取得实质性进展。改革与管理综合考评名列全省第一。189个单位、11729人纳入部门预算管理，编制范围覆盖了市直行政事业单位；全市纳入国库集中收付单位137家，占市直预算单位的76%，一级全额预算单位全部纳入了改革范围；政府采购在完成机构建设的同时，强化制度约束，全年纳入集中采购376次，采购资金9745.9万元，节约资金1149万元，节约率10.6 %；收支两条线改革进一步深化，政府非税收入管理更加规范。

机构改革不断深化。围绕转变政府职能，提高行政效率，全面推进市、区、乡镇机构改革。市级撤并、整合、更名机构8个，调整人员273名，市直事业单位综合配套改革和依法规范管理有新的突破；区级党政机构均减少1个，事业机构均减少18个，事业单位编制减少281名，减幅达75%；乡镇机构均减少2个，人员定编定岗工作有序进行。长港农场改革成效明显。商贸、粮食、供销等流通领域的改革全面推进。

（五）社会事业不断进步。坚持以科学发展观为指导，在发展的基础上增加建设和谐社会的物质基础，在构建和谐社会目标上加快发展，是新形势下政府工作新的决策选择。

科技教育事业长足发展。全市高新技术产业增加值为11.15亿元，比上年增长22.5%；省级高新技术企业达26家，比上年增长8.3%；专利申请首次达到106件，比上年增加1倍。教育事业协调发展，“普九”达标通过省级复查，鄂州高中通过“全省示范高中”验收。办学条件不断改善，教育信息化步伐加快，全市高考取得新成绩。

文化卫生计划生育工作成效显著。文化体育、广播电视事业繁荣发展，凤凰广场荣获全国特色文化广场，广电中心大楼竣工并投入运营。卫生改革与发展取得新成效，实施7个公共卫生国债建设项目，总投资2600万元，艾滋病、血吸虫病等重大疾病的防治工作取得新突破。人口与计划生育工作取得新进展，人口出生率比省定责任目标低4.99个千分点，平抑新生儿性别比失衡工作取得新的成效。

社会保障工作不断加强。全市城镇净增就业岗位1.9万个，启动了下岗失业人员小额担保贷款工作。“两个确保”、“一个低保”工作得到巩固。全年发放下岗职工生活费1101万元，发放城市最低生活保障费1599万元，拨付救灾救济款418万元，安排农村五保户生活费440万元，发放住房公积金政策性贷款5000万元，帮助了部分中低收入职工改善了居住条件。一年来，社会保障工作全面启动，困难群体生活条件得到了明显的改善。

民主法制建设深入进行。2004年共办理人大代表议案和建议、政协委员提案258件，办复率达100%。积极推进依法治市，“四五”普法工作深入开展，政府法制建设成效明显，审计、监察工作得到加强。认真落实社会治安综合治理责任制，严打整治斗争取得成效，刑事发案率比上年下降10%。

在取得上述各项成绩的同时，对老区贫困地区的扶持力度不断加大。安全生产被评为全省先进单位，社区建设取得新的进展。民族宗教、外事侨务、对台、信访、物价、统计、档案、人防、消防、老龄、气象、农机和国防教育、民兵建设等工作都取得了新的成绩。

各位代表，过去一年的成绩来之不易，是全市人民团结奋斗的结果。借此机会，我谨代表市政府向在各个岗位上辛勤劳动、作出贡献的广大干部群众，向各位人大代表、政协委员，向各民主党派、工商联、无党派人士、人民团体和社会各界人士，向驻军指战员、武警官兵以及所有关心支持我市建设的港澳台同胞、海外侨胞、国际友人表示崇高的敬意和衷心地感谢！

回顾过去一年，我们也清醒地看到，政府工作中还存在一些薄弱环节，经济社会生活中还存在一些突出的矛盾和困难。主要有：一是农业基础脆弱，农业增效、农民增收的长效机制尚未形成；二是产业结构不尽合理，经济发展的瓶颈制约问题仍然突出，部分企业经济效益下滑；三是就业再就业压力大，社会保障体系不健全，部分下岗职工、城镇低收入居民和贫困地区群众的生活还比较困难；四是发展环境还不够宽松，“三乱”现象屡禁不止，有的部门利益严重，办事效率低下；五是社会治安形势严峻，人民群众反映强烈；六是政府自身建设尤其是行政能力建设、廉政建设、作风建设，还需要进一步加强。对这些困难和问题，我们一定要高度重视，采取得力措施，努力加以解决。

二、2005年工作安排

今年是全面贯彻党的十六届四中全会精神的第一年，是落实“十五”计划的最后一年，也是衔接“十一五”计划的基础年。做好今年的工作，具有十分重要的意义。今年政府工作总体要求是：以邓小平理论和“三个代表”重要思想为指导，认真贯彻党的十六大、十六届三中、四中全会精神，全面落实科学发展观，紧紧把握融入武汉城市圈的战略机遇，坚持以发展为主题，以项目建设为重点，以人民群众根本利益为出发点，进一步加大改革开放、结构调整和环境优化力度，进一步加快推进新型工业化和农业产业化，着力解决经济社会发展中的突出矛盾和关系群众切身利益的突出问题，努力构建和谐社会，促进经济社会全面协调可持续发展。全市经济社会发展的主要预期目标是：地区生产总值160亿元，增长12%；全社会固定资产投资52.3亿元，增长18%；社会消费品零售总额61.1亿元，增长12%；实际利用外资6520万美元，增长10%；外贸出口2.93亿美元，增长10%；财政收入12亿元，增长14.8%；城镇居民人均可支配收入8020元，增长7%；农民人均纯收入3414元，增长5.6%；城镇新增就业2.1万人，城镇登记失业率控制在4.5%以内，人口自然增长率控制在5‰以内。

确定上述目标，主要是基于以下考虑：一是宏观经济环境有利。中央作出的宏观调控政策，理顺了市场秩序，避免了盲目竞争，对鄂州是一次难得的发展机遇。有利于我市冶金、建材等支柱产业加快技术改造、抢占市场空间。只要我们在调控中保持奋发向上的精神和旺盛的斗志，我市经济就一定能在调控中取得更大的发展。二是经济发展基础较好。近几年来，随着我市“一化带三化”战略的整体推进，我市经济增长的亮点频出，一批又一批重大项目相继投产或开工，为加快发展奠定了较好的基础；城市规模的扩大、基础设施建设的加强、企业“宁静日”制度的实施、机关作风的转变，使发展环境进一步改善，这些都为经济社会发展创造了良好的条件。三是内在发展动力增强。鄂州经济社会发展取得的新成就，极大地增强了全市人民的信心，保持共产党员先进性教育活动的开展也势必激发广大干部群众的斗志，为加快发展提供强大精神动力。民营经济的发展和国有企业改制改革的深入进行，为经济发展创造了强大的内在动力。因此，只要我们不断强化机遇意识和责任意识，按照科学发展观的要求，既保持较快增长速度，又注重质量、效益提高；既抓好经济建设，又重视社会事业发展；既努力增强综合实力，又高度关注富民问题，就一定能够完成和超额完成今年的各项任务。

（一）加强项目建设，推进新型工业化进程

工业化是经济现代化的基础，是财税增长的主体。率先发展工业，是鄂州的现实基础和长远需要所决定的。因此，坚持“工业兴市”，坚持以项目为中心、加速推进新型工业化进程，是政府工作的首要任务。

深入实施“三二三”工程。继续集中力量抓好一批事关全市大局和长远发展的重大项目，认真组织实施“三二三”工程，逐步培养一批在全省乃至全国叫得响、吃得开、立得住的明星企业、名牌产品。今年计划安排固定资产投资52亿元，重点支持三个税收过3亿元的大型企业、20个税收过千万元的企业、30个科技含量高具有发展前途的重点项目的建设。加大扶持力度，确保武钢500万吨球团矿厂今年投产。促进鄂钢6米焦炉干熄焦工程、制氧机改造、5号连铸机和葛店开发区浩信药业、铅酸蓄电池等项目投产；促进扬子江药业搬迁、四环药业、世纪新峰二期、鸿泰钢铁二期、枫树线业四期扩建、易初莲花购物中心等项目开工。认真做好鄂州电厂二期的前期工作，争取国家核准并开工建设。继续实行项目领导责任制，对重大项目实行市级领导挂点制度，确保项目建设顺利进行。

加快特色经济板块建设。按照“积极引导、相对集中、龙头带动、群体发展”的原则，依托我市产业、区位、资源等优势，立足现有基础，瞄准市场需求，规划发展一批有一定规模、优势突出、辐射力强的特色经济板块。积极围绕“三个千万吨”目标做大做强我市的支柱产业，即再经过几年的努力，在鄂州实现一千万吨钢铁、一千万吨水泥、一千万吨矿产品的生产能力，使鄂州真正成为鄂东冶金建材走廊的“黄金地段”。与此同时，不断改善葛店高新技术开发区、红莲湖旅游度假区、花湖民营经济开发区、梁子湖（岛）生态旅游度假区的软硬环境，吸引金融资本、民营资本、科技资本向园区集中，使四个各具特色的开发区充满生机和活力，成为我市新的经济增长点。充分发挥白浒镇铸造业、燕矶金刚石刀具、梁子湖非金属矿业等产业现有的集聚优势，依托项目加速产业优势的形成，发展园区经济，建设产业集群。

大力发展高新技术产业。围绕生物医药、新材料、电子信息等重点行业，大力推进高新技术产业化，力争高新技术产业产值比上年增长20%。着力建好城区科技企业孵化器，争取建成一个省级科技创新示范区。鼓励企业技术创新，形成一批具有自主知识产权的科技成果，确保专利申请量比上年增长20%。多方筹措资金，建立“科技型中小企业技术创新基金”，用于实施重大科技项目和技术创新。以湖北莲花山计算机视觉和信息科学研究院的建设为重点，积极扶持民营科研机构的发展。

（二）提高农业综合生产能力，建立农民增收长效机制

坚持以农民增收为中心，以农业增效为重点，进一步提高农业综合生产能力，加速推进农业结构调整和农业产业化经营，逐步建立农民增收的长效机制。

认真落实党和国家的农村政策。今年党中央国务院和省委省政府相继颁布了两个一号文件，党和国家对“三农”问题作了明确有效的政策规定，把这些政策落实到千家万户，让广大农民享受党和政府的惠农政策是各级政府的重要职责。从今年开始，我市实行“一免三补”的惠农政策，即在全市免征农业税，对种粮农民实行直接补贴、良种补贴和农机具购置补贴。我在这里郑重宣布：由70万农民长期负担的农业税从今年起全部取消，农民种田再也不交税了。各级政府必须切实贯彻中央和省委两个一号文件精神，切实做好农村土地第二轮延包工作，加强督办检查，使党和国家政策真正惠及到千家万户。

加强农业基础设施建设。增加农业发展后劲，提高农业抗御自然灾害能力，必须大力加强农业基础设施建设。现有农业基础设施大部分已经老化，必须建立完善农业基础设施投入体制，多方筹措资金，重点解决抗旱排涝的泵站、渠道、农田水利和病险水库的建设以及农村塘堰改造。加强农村水、电、路、通讯等基础设施建设，加快实施农村“五改”（改水、改路、改厕、改圈、改厨）、“三建”（建沼气池、建庭院经济、建生态家园）工程，改善农民的生产生活环境。必须千方百计地筹集农业基础设施建设资金，积极向上争取国家建设项目，市政府及主管部门将逐年加大投入，并加强专项资金的投入监管力度。必须改革农业基础设施管理制度，使农业基础设施按市场化的方式运营，并能在运营中保值增值，建立农业基础设施投入、管理、运营的良好运行体系。

继续抓好农业基地建设。按照“城郊型”农业定位，壮大水产、蔬菜、畜禽、果木四大支柱产业，加快50万亩无公害水产板块基地建设，力争把杜山——长港——东沟30公里连片水产带办成全省水产基地的精品。高标准推进无公害蔬菜基地建设，形成新庙——燕矶——杨叶20公里蔬菜产业带。充分发挥湖北鄂州原种猪场和武昌鱼猪场等12家万头猪场的带动作用，加大优质“三元猪”推广力度，加强重大动物疫情的防治工作，使我市尽快成为全省优质“三元猪”的良种基地、扩繁基地、出口基地。抓好10万亩胡柚基地的续建工作，加快东佛苗圃基地、杜山黄金梨基地、蒲团西甜瓜基地建设步伐。广泛开展“农业实用技术落实年”活动，加强先进适用农业技术和优良品种的普及和推广，实施科技兴农战略，建成优质稻基地20万亩。

突破性发展农产品加工业。发挥我市现有农业资源、资产、品牌优势，促进农产品精深加工上规模、上水平、上档次。围绕“武昌鱼”和“梁子湖”品牌，整合水产资源，做强做大水产品加工业，使我市成为全省水产品加工和出口创汇基地。加大对市广源米业有限公司、嘉禾粮油有限公司、凤凰薯业有限公司等企业的扶持力度，使保健米、保健油、优质淀粉生产逐步形成规模，争创粮油加工知名品牌。根据国际通行的卫生标准和质量标准，推进蔬菜、畜禽、果木由半成品加工向精深加工转变，提高产品档次，增强市场竞争力。加快以蟠龙大市场为龙头的农业流通网络建设，培养和扶持更多的科技人才和农产品经纪人，促进我市优质农产品加工的快速发展。

用减少农民的办法富裕农民。我市现有耕地面积54.5万亩，农民人均0.74亩。国际经验和改革开放20多年的实践证明，有限的土地资源是不可能让农民获得真正的富裕。从我市的实际出发，富裕农民必须减少农民。积极鼓励农民进城创业立业，市政府已经出台了全市户籍制度改革办法，放低农民进城入户的门槛，只要他们具有一定数量的城市资产，具有一定数量的资本产业，都允许农民进城落户；积极鼓励农民参加各类培训，提高科技素质，增强风险意识，主动适应市场经济发展的要求；积极鼓励农民外出务工，让更多的农民外出从事二、三产业，直接增加农民收入；积极鼓励外出务工农民回乡创业，反哺农业，促进农业良性发展。加快农村小集镇建设，使更多农民离土不离乡，让农民在农村小集镇就业创业，促进城乡的发展，实现社会的稳定。

（三）加快城市建设步伐，改善经济发展环境

围绕把鄂州建设成为环境优美、设施完善、生态和谐、市场繁荣、特色鲜明的山水园林城市，加强城市规划、建设和管理，努力塑造鄂州城市新形象。

加强城市规划的修编和管理。以建设和谐鄂州为目标，以融入武汉城市圈为契机，拓宽城市规划的视野，既要体现武汉城市圈发展的大概念，更要体现鄂州经济圈发展的新需要。充分展示城乡统筹发展、经济社会全面进步、人民安居乐业的理想目标，建设新鄂州。为此，在总体规划的修编上，按照西连武汉、东接黄石和城乡统筹发展的总思路，形成以主城区为中心，以华容——葛店、花湖——杨叶和梁子湖三个组团为支撑的“一主三辅”的城镇体系。中心城区要积极推进控制性详规编制工作，力争控制性详规覆盖率达85%以上。华容——葛店的西部城区要依托武汉，实施西进战略，实现葛店开发区与东湖开发区对接，葛华城区与葛店化工城对接，红莲湖度假区与武汉市场对接，使之在融入武汉的进程中，完善功能，加快发展；花湖——杨叶的东部城区要接轨黄石，实施东扩战略，使东部城区的几个乡镇与黄石实现城市发展、产业转移、市场布局、基础设施共用的一体化格局；梁子湖区要构建与武汉、黄石相联的便捷的交通网络，实现人流、物流、信息流、产业流一体化，谋划新的城镇发展思路，在发挥资源优势、打造生态旅游品牌的过程中，保护环境，形成特色。加强规划管理，创新规划决策机制，确保城市建设步入规范化轨道。

加快推进城乡一体化进程。按照城市建设总体规划和融入武汉城市圈的要求，积极推进城乡一体化，构筑“一主三辅”之间的交通网络体系，强化城镇相互之间的互动，对于建设现代化的鄂州非常重要。认真做好中心集镇的规划修编工作，使详细规划的覆盖率达到100%。引导农民遵守规划移民建镇，使农民建房向规划化的集镇管理方向迈进。推进城镇化对改变农村落后面貌，实现农民向城镇居民转变，提高农产品商品率，为政府提供公共产品建设具有重大的意义。必须把城镇化作为建设富裕型小康城市的重要措施抓落实；把它当成是为广大农民生活找出路、为农村建设找方向、为农业发展找后劲的重要任务抓落实；把它作为实现城乡统筹发展、建设和谐鄂州的重要环节抓落实。

加快城市基础设施建设步伐。以争创“楚天杯”为目标，不断加快城市建设步伐。今年城区基础设施建设重点实施“6436”工程，即：新、续建寿昌大道、滨湖东路、滨湖南路等6条城市道路；新、续建泽林铁路立交桥、樊口大桥等4座桥梁；启动和新建寒溪（西山）、雨台山等3个景点；改造十字街、熊家巷等6条老城区道路。农村基础设施建设以道路连片成网为重点，实施6项公路工程，即：鄂黄公路路面工程、63.9公里通乡油路、程潮进矿公路路面、太友公路路面、葛湖公路路基路面、106国道（泽林——分水岭）改建工程，完成梁子湖航道疏浚二期工程和鄂州一级中心客运站工程。大力发展住宅建设，促进房地产业健康发展，实现全年开工面积40万m2，竣工面积30万m2。加强物业管理，提高市民居住质量。

不断提高城镇管理水平。以整洁有序为重点，坚持标本兼治、重在治本的指导思想，加大城管执法力度，有步骤地将严管制度和措施向背街小巷和城乡结合部延伸。大力整治城市“牛皮癣”，严厉制止乱搭乱建、出店经营，加大城中村的管理力度。市直各部门和各社区要创造性地办好市民学校，有计划、有步骤、有目的地培训市民，提升市民的整体素质，树立鄂州人的新形象。继续完善市容协管员门前“三包”工作，不断增强市容管理的整体联动能力。不断完善市政设施，加强破损路面维修，认真做好市容环卫保洁工作。

强化资源及生态保护。完成土地利用总体规划修编，不断提高节约和集约用地水平。坚持开发和节约并举，把节约放在首位，依法保护耕地、水、森林、矿产等资源。按照分类指导、分区推进、分步实施的要求，全方位推进生态示范市创建工作。切实加强新上项目的环境评价和监管力度，坚决地实施环境评价一票否决权。加大建材、冶金行业结构性污染整治力度，加快解决城区环境污染问题。加大沿江沿湖综合治理力度，抓好梁子湖生态保护工程、环洋澜湖绿化工程、洋澜湖截污工程等建设。

加快服务业发展步伐。积极启动住房、教育、信息等新型消费，统筹发展运输、仓储、包装等物流经济。大力发展连锁经营、电子商务等现代流通方式，加快建立多种形式的物流配送体系。主动积极地对接武汉都市旅游圈，认真做好中国优秀旅游城市的复核工作，加快编制天平山等重点旅游景点建设规划，推动旅游业健康、持续、快速发展。

（四）加快民营经济发展，增强经济发展后劲

大力发展民营经济是加快我市发展步伐、增强发展后劲的必然要求；是提升经济总量、壮大经济实力、建设富裕型小康城市的必由之路；是不断丰富物质基础、以人为本建设和谐鄂州的基本保障。

放宽民营经济投资和创业条件。按照“非禁即入”的原则，进一步放宽市场准入政策，继续清理不适应民营经济发展要求的过时规定，建立一套有利于民营经济发展的政策体系，进一步降低准入门槛，放宽企业集团登记条件，放宽经营场所限制，规范行政许可行为。鼓励和支持全民创业，让更多有经营天才的商界奇人、业界巨子脱颖而出，为鄂州发展献计出力，增砖添瓦。

鼓励民营企业做大做强。对民营经济的发展，既要鼓励人人创业、个个显露身手，但更要鼓励他们做大做强。市场经济是竞争经济，只有强大的实力才能应付残酷的市场竞争。支持民营经济做大做强，就是对民营企业要公平税费政策、公平使用政府扶持资金、公平享受金融资本、公平征用土地；鼓励他们开展技术创新，鼓励他们由作坊式企业向现代企业转变，鼓励他们由家族型向公司型转变，鼓励他们走外向型企业发展之路，鼓励他们从产业资本向跨行业转移。

坚决维护民营经济的合法权益。整治部门的“三乱”行为，斩断伸向企业的黑手，坚决地规范各种执收执罚行为，维护民营企业合法权益。但是，对于少数民营企业制售假冒伪劣商品、非法使用童工、不执行最低工资标准、任意克扣工资、侮辱劳动者人格以及其它涉黑涉黄涉毒等非法行为也决不姑息迁就。同时，加快建立健全民营经济社会化服务体系，建立健全民营企业合法权益保障体系，建立健全民营企业科技进步和人才培养引进体系，使我市民营经济发展跃上新的台阶。

营造尊重纳税人的良好氛围。必须在全市形成“投资者是上帝、引资者是功臣、服务者是公仆、干扰者是罪人”的共识。必须认识到纳税人是我们的“衣食父母”，是纳税人直接创造了社会财富，是纳税人提供了就业岗位，是纳税人为政府的公共支出提供了税收。因此，必须充分地保护纳税人的合法权益，严厉地打击损害纳税人利益的不法行为。不仅如此，政治上要关心他们的成长进步，支持他们上党校或参加行政学院学习，在入党、评模、当先进方面都要一视同仁，使纳税人更加自觉地、满腔热情地投身我市的经济建设。

（五）主动融入武汉城市圈，不失时机地抓住发展机遇

建设武汉城市圈是省委省政府呼应中央关于“中部崛起”的重大战略决策。这一决策在今年省十届人大三次会议上省人大代表作了热烈的讨论，目前“1+8”的概念深入人心，它已成为全省人民的共识。我市是离武汉城市圈核心城最近的一个城市，如何利用这一良好机遇，加快鄂州的发展，是摆在全市人民面前的重大选择。

建立资源共享机制，实现乘数效应。我市与武汉城市圈中的武汉、黄石两大城市是地相连、路相通、业相接、人相融，具有得天独厚的区位优势，充分利用好两大城市的交通、给排水、电力、通讯、市场等硬件设施，主动地进行规划、交通、市场、产业的对接，就能迅速改换我市的发展环境；充分利用两市的人才、技术、文化、思想观念等智力资源，就能迅速地增强我们的发展后劲；充分利用大城市产业转移的机遇，使我市形成与两市互为“前店后厂”的工业布局、互通有无的商品交换市场、互相支撑的经济共同体，就能实现经济发展中的“乘数效应”。

调整思路和决策，形成资本洼地。在融入武汉城市圈过程中，我们占有天时地利，但如何占有人和，则需要我们自身的努力。融入武汉城市圈必须创新思路、科学决策，围绕我市经济发展的“一主三辅”的“四个经济圈”做文章，即以主城区50平方公里为圆圈的经济圈，以华容区、葛店开发区、红莲湖度假区和武汉市的洪山区、东湖开发区、葛店化工城为圆圈的经济圈；以鄂城区的花湖、杨叶、燕矶、汀祖、碧石和黄石为圆圈的经济圈；以梁子湖区、梁子湖旅游度假区和武汉市的江夏区、黄石市的大冶市为圆圈的经济圈。在发展上实行非均衡战略，条件好的优先发展；在政策上创造更为宽松的环境，营造更多的商机，使资本源源不断地流向我市，促进共同发展。

牢固树立机遇意识，捷足先登。融入武汉城市圈，发展鄂州经济，虽然我们占有先天优势，但如果不努力抓住目前的机遇，我们只会靠着金山银山没饭吃。为此，树立机遇意识、抢抓机遇非常重要。发展不够不仅是全国、全省的问题，也是武汉城市圈内“1+8”都面临的问题，争投资、争项目、争人才将在相当长的时间内存在。那么在发展自己的问题上一点不能含糊，就是按照小平同志讲的“抓住机遇，发展自己，关键是发展经济”。

（六）积极扩大对外开放，提高招商引资质量和水平

努力适应对外开放的新形势，始终坚持引进外资与引进内资并重，进一步提高利用两个市场、两种资源的能力。

创新招商引资方式方法。积极推行产业招商、项目招商、园区招商、资源招商、小分队上门招商等多种形式招商，继续以日韩、欧美等发达国家和港台、沿海等发达地区为重点，力争在引进大公司、大集团上取得突破。充分利用鄂港经贸洽谈会、厦门投洽会、深圳高交会、广交会等平台，精心组织策划项目推介，确保招商活动取得实效。结合行业、产业和企业结构调整，突出冶金、建材、生物医药等优势产业及相关项目，推动配套项目投资、行业产业链延伸。葛店开发区要充分利用“葛店·中国药谷”品牌资源，增强招商引资的龙头带动作用。继续坚持、完善和落实好企业“宁静日”制度，使其逐渐成为我市招商引资的一块牌子。加强对重大签约项目的跟踪、服务力度，提高项目履约率和资金到位率。进一步落实招商引资优惠政策，促进在外创业立业成功的鄂州籍老板回家创业发展，报效桑梓。必须严格执行考核奖惩制度，对招商引资实绩突出的单位和个人进行重奖，力争今年招商引资取得新突破。

优化外贸出口商品结构。适应出口退税机制改革，调整出口结构，提高产品质量，增强市场竞争力。纺织、服装等产品在巩固日本、韩国、东盟等传统市场的同时，积极开拓欧美、中东及非洲等新兴市场。努力提高机电产品和高新技术产品的档次和技术含量，加快适应国际市场的新变化、新特点，不断扩大国际市场份额。对美阳服装、华源电子、多佳贸易、泰德富华、长江粮油等出口大户，继续实行挂点联系责任制，主动热情地为重点企业扩大出口创造条件，确保外贸出口有新的增长。

扩大对外经济技术合作。鼓励和支持有条件的企业对外投资，采取合资、独资、联营等形式开展跨国、跨省经营，开发市外资源，带动我市产品、设备、原材料等商品出口和劳务输出。认真研究不同国家、不同地区的发展水平和市场状况，有针对性地实施“走出去”战略。多方搜集劳务供求信息，加强劳务输出与合作，为解决农村剩余劳力和下岗职工再就业服务。

（七）不断深化各项改革，增强经济发展活力

改革是经济发展的不竭动力。按照建立和完善社会主义市场经济体制的总体要求，进一步清除制约生产力发展的体制性障碍，激发经济主体的创造热情。

继续深化国有企业改革。已经列入改制计划的市属重点企业方案的审批率确保达到100%，已改制企业的职工安置到位率确保达到95%以上。对已完成改制的企业继续搞好跟踪服务，使其尽快实现经营方式和经济增长方式的转变，尽快进入健康良性的发展轨道；对尚未完成改制任务的企业，今年继续进行改制。引导改制企业实现多途径资产变现，把推进企业改制与招商引资紧密结合起来，大力支持困难企业在符合城市总体规划的前提下利用土地进行开发，筹措改制资金。切实抓好特困企业投保安置工作。对特困企业改制，继续坚持市级领导挂点责任制、驻企工作组帮促责任制、企业主管部门目标责任制和市改制办协调、督导责任制，加快改制工作步伐。

继续深化财税金融改革。不断地改善支出结构，更好地兼顾公平与正义；加强财政资金的管理，更好地做好社会保障工作，是财政改革的基本方向。建立和完善以国库单一账户体系为基础、资金缴拨以国库集中收付为主要形式的财政资金管理制度，坚决取消预算单位自行开设的银行账户。凡确定实施国库集中收付的单位，所有资金必须全部无条件纳入国库单一账户体系统一管理，坚决杜绝私设“小金库”现象。完善政府采购操作规程和运行机制，将政府采购融入部门预算、集中收付和收支两条线管理改革之中，整体联动，配套推进。认真落实收支两条线管理制度，结合非税收入收支管理制度改革，将行政事业性收费、政府性基金、罚没收入等全部纳入财政预算管理，做到单位上缴收入不再与其支出挂钩。坚持依法治税，强化税收征管。加强和改进金融监管，保证金融平稳运行。积极支持金融部门深化改革，调整优化信贷结构，完善信用担保体系，加大对重点行业、企业、产品和项目的信贷支持力度，积极支持金融部门向梁子湖区延伸机构。支持农村信用社改革，改善农村金融服务。

继续深化行政管理体制改革。深化政府机构改革，理顺行政管理体制，明确职能分工，大力精简机构和人员，努力解决层次过多、职能交叉、权责脱节和多重执法等问题，坚决纠正政府利益部门化的问题。按照政事分开原则，推进事业单位管理体制和人事制度改革。以分配方式的改革为突破口，不断探索事业单位改革的新思路。建立人才最低收入保障机制，千方百计吸引人才、留住人才。严格执行市委、市政府出台的乡镇机构改革的系列规定，依法依规推动改革顺利进行。

（八）统筹发展各项社会事业，建设和谐鄂州

在加快经济发展的同时、更加注重社会事业的发展，在加快物质文明建设的同时、更加注重精神文明建设，逐步使我市走上生产发展、生活富裕、社会和谐的发展道路。

认真做好科教文卫计划生育工作。深化科技体制改革，增强科研机构的创新能力和面向市场的活力。把教育放在优先发展的地位，进一步促进各级各类教育协调发展。加强未成年人和大中专学生的思想道德建设。健全农村义务教育投入保障机制，确保教师工资按时足额发放，确保农村适龄儿童享受九年制义务教育。大力发展职业教育，加强职业技能培训。抓好教师队伍建设，提高教学质量和水平。采取得力措施，争取早日实现鄂州大学专升本。推进和规范社会办学，促进民办教育健康发展。继续加强公共卫生体系和医疗基础设施建设，抓好血吸虫病、艾滋病、结核病、肝炎等重大疾病的监控和防治，积极稳妥地推进新型农村合作医疗制度试点，逐步建立由政府组织和引导，农民自愿参加，个人、集体和政府多方筹资，以大病统筹为主的农民医疗互助共济制度。进一步加大人口与计划生育工作力度，落实农村部分计划生育家庭奖励扶助政策，坚决打击非法鉴定胎儿性别和选择性别终止妊娠的行为，确保出生人口性别比控制在一定的范围内。探索充分利用行政、社会、市场三方面力量开展文化体育活动的新路子，进一步净化农村文化市场，扶持农村文化中心户建设。进一步发展文学艺术、新闻出版和广播影视业，重视发展哲学社会科学，为人民群众提供更多的优秀精神产品。

积极完善社会保障体系。认真落实企业退休人员基本养老金政策，确保企业离退休人员养老金社会化发放面和发放率达到100%。积极探索机关事业单位养老金社会化发放新举措，不断提高社会化发放水平。进一步扩大社会养老保险覆盖面，加大力度提高非公有制企业的参保率。进一步完善社会保险登记、申报、核定制度，努力提高社会保险接续率，减少参保人员流失。全面加强失业、医疗、工伤、生育保险管理，推进保险监测、预警和评估体系建设，积极发挥商业保险的补充作用。完善以城乡特困群体救助为主要内容的社会救助体系，探索解决失地农民长远生计问题的办法。调整最低工资标准，督促企业制定工资支付办法，严格执行欠薪预警制度，切实保护职工合法权益。进一步推行和完善住房公积金制度，改善广大职工的居住环境和条件。

切实做好就业再就业工作。坚持政府指导、市场推动的就业方针，健全和完善就业服务制度，统筹城乡就业工作。大力发展劳动密集型产业、非公有制经济和中小企业，多途径开辟就业渠道。认真落实扶持就业再就业的各项政策，抓好“4555”人员社保援助、灵活就业人员社保补贴、社保补贴拨付办法和小额担保贷款办法“四项改革”试点工作，解决好困难地区、困难行业、困难群体的再就业问题。

大力整治社会环境。坚持“打防结合、预防为主”的方针，加强社会治安综合治理，提高大案要案侦破水平，依法严厉打击各种刑事犯罪、经济犯罪和黑恶势力，特别是要坚决地打击企业和建筑市场中强迫交易、敲诈勒索以及黑恶势力插手工农关系、劳资纠纷、医疗纠纷、各种安全事故等违法行为，维护正常的生产生活和医疗秩序；坚决地整治校园周边治安环境，确保校园师生的安全，维护正常的教学秩序；坚决地清理整治营业性网吧，确保青少年健康成长；坚决地防止和纠正损害群众合法权益的行为，维护群众的合法利益；坚决地整顿规范市场经济秩序，积极开展食品、药品、农资、烟草等市场的专项打假活动；坚决地贯彻落实国务院信访工作条例，及时解决群众反映强烈的热点难点问题。高度重视和切实抓好安全生产，努力实现安全生产形势的进一步好转。与此同时，加强对国家公务员的公仆意识教育，加强对执法人员的服务意识教育，加强对医生教师的职业道德教育，认真地学习贯彻《公民道德建设纲要》，为建设和谐鄂州提供精神保障。

切实加强社区建设。随着城市化进程的不断加快，不仅是城镇居民进入社区，而且很多企业、国家机关事业单位的退休、下岗分流人员，农村进入城镇的居民、外来务工人员都将纳入社区管理。因此，新形势下社区建设具有更大的意义。必须加强社区建设的领导，健全网络，切实解决好社区建设中的资金、场地、人才问题，使社区成为政府联系城镇居民的桥梁和纽带，成为解决各种社会矛盾的“缓冲区”，成为建设和谐社会的“示范区”。

在做好上述各项工作的同时，加大对老区、贫困地区的扶持力度，改善困难群众的生产生活条件。充分发挥外事侨务部门的纽带和桥梁作用，进一步扩大对外交往与合作。加强国防教育，做好民兵预备役工作。扎实推进经济普查，大力发展助残事业，认真做好人防、统计、物价、气象、农机、民族宗教、档案、保密、老龄、消防等工作。

各位代表，心系群众、关心群众是各级政府应尽的职责。今年，市政府在认真抓好经济和社会发展各项工作的同时，还将为全市人民办好八件实事：一是筹措240万元对农村1万名特困人口实施救助；二是筹措300万元对城乡特困群体实施大病医疗救助；三是筹措400万元对城乡贫困群体子女实施教育资助；四是筹措470万元全面解决1.7万农村居民饮水困难；五是实现1万户城区居民使用天然气；六是筹措600万元完成农村中小学校D级危房改造；七是筹措60万元对6000名农村劳动力进行转移培训；八是筹措260万元为下岗失业人员提供600个公益性岗位。

三、加强政府自身建设

按照“行为规范、运转协调、公正严明、廉洁高效”的要求，切实加强政府自身建设，以开展保持共产党员先进性教育活动为先导，牢固树立马克思主义世界观、人生观、价值观，牢固树立正确的权力观、地位观、利益观，增强全体公务员的服务意识，提高行政能力和管理水平。

坚持科学民主决策。建立健全公众参与、专家论证和政府决定相结合的公开透明的行政决策机制，充分发挥民主党派、工商联、无党派人士在决策中的重要作用，不断推进决策的民主化、科学化、规范化。进一步完善专家咨询制度，实行决策前的咨询论证制、决策中的集体票决制和决策后的绩效评价与责任追究制，实现由经验决策向科学决策的转变；制定和完善人民群众建议征集制度，继续大力推行对涉及公众利益的重大事项社会公示制和社会听证制，充分体现群众意愿，实现由封闭式决策向开放式决策的转变；大力实施阳光政务，继续推进政务公开，不断提高政府工作的透明度。

坚持依法行政。严格按照法定权限和程序行使权力，依法规范政府及政府各组成部门的行为。全面完成“四五”普法工作。深入贯彻落实《行政许可法》，认真做好行政许可项目的清理和公示工作。进一步理顺行政执法体制，相对集中行政处罚权，切实解决多头执法、多层执法、执法扰民以及部门利益化等群众反映强烈的问题。继续推行行政执法责任制，严格执行错案责任追究制，认真抓好行政复议工作。加强民主政治建设。自觉接受人大及其常委会的法律监督，及时报告工作，落实各项决议、决定；自觉接受政协的民主监督，适时向政协通报情况，听取政协委员意见；认真办理人大代表议案、建议和政协委员提案，支持民主党派参政议政，欢迎人民群众与新闻媒体对政府工作进行舆论监督。

加强廉政建设。积极构筑反腐倡廉的制度体系，在全市形成以廉为荣、以贪为耻的良好社会风尚。不断深化以行政审批制度、财政管理制度、投资体制、干部人事制度“四项改革”为重点的相关改革；不断完善以经营性土地使用权出让、建筑工程项目公开招投标、产权交易、政府采购“四项制度”为重点的约束机制；不断规范以行政服务中心、综合招投标中心、国库集中收付中心、经济发展环境投诉中心“四个中心”为重点的平台建设；不断拓展以政务、厂务、事务、村务“四个公开”为重点的监督渠道，从源头上防治腐败。加强行政监督和审计监督，全面实施领导干部经济责任审计制度。以解决群众反映的突出问题为重点，坚决纠正损害群众利益的不正之风，加大对违纪违法案件的查办力度，敢于碰硬，决不手软，以反腐倡廉的实际成果取信于民。

提高政府工作效能。大力建设服务型政府，坚持以人为本，把群众呼声当作政府工作的“第一信号”，把为人民群众谋利益作为“第一职责”，把优化发展环境作为政府工作的“第一着力点”。在履行好政府宏观经济调节和市场监管职能的同时，进一步强化社会管理和公共服务职能，注重提高应对突发事件的能力。创新工作方式，加快电子政务建设步伐。加强政府工作人员的教育和管理，推动公务员队伍提质提效提速，建设一支政治坚定、业务精通、作风优良、纪律严明、恪尽职守、善于服务、甘于奉献的公务员队伍，切实解决公务员队伍中不作为、乱作为和慢作为的问题。大力弘扬求真务实的作风，精简文山会海，减少接待应酬，集中精力和时间，深入基层、深入实际，研究经济社会发展中的各种困难和矛盾，解决基层和群众关心的热点难点问题。坚决反对形式主义、作表面文章，坚决反对弄虚作假、虚报浮夸。围绕市委的决策部署和人代会确定的任务，完善目标责任管理考核体系和办法，加强对重大工作事项的督办检查，确保各项工作部署落到实处。

各位代表，今年是“十五”计划的最后一年，加快鄂州改革开放和经济社会发展的任务十分艰巨。让我们高举邓小平理论伟大旗帜，以“三个代表”重要思想为指导，在省委、省政府和市委的正确领导下，依靠和团结全市人民，坚定信心，脚踏实地，开拓进取，扎实工作，为全面和超额完成今年的各项工作目标、建设富裕型小康城市而努力奋斗！
